# Real-World Clinical Outcomes for Patients with *EGFR* and *HER2* Exon 20 Insertion-Mutated Non-Small-Cell Lung Cancer

**DOI:** 10.3390/curroncol30080515

**Published:** 2023-07-25

**Authors:** Kelly Li, Ian Bosdet, Stephen Yip, Cheryl Ho, Janessa Laskin, Barbara Melosky, Ying Wang, Sophie Sun

**Affiliations:** 1 Department of Medical Oncology, BC Cancer Agency Vancouver, Vancouver, BC V5Z4E6, Canada; 2 Cancer Genetics Laboratory, BC Cancer Agency Vancouver, Vancouver, BC V5Z4E6, Canada

**Keywords:** NSCLC, exon 20 mutations, *HER2*, *EGFR*, targeted therapy, real-world outcomes

## Abstract

(1) Background: Exon 20 insertion mutations (ex20ins) in *EGFR* and *HER2* are uncommon driver mutations in non-small-cell lung cancer (NSCLC), with a poor prognosis and few targeted therapy options, and there are limited real-world data. Here, we report the clinicopathologic features and outcomes for patients with ex20ins NSCLC across British Columbia, Canada. (2) Methods: NSCLC patients with ex20ins in *EGFR* or *HER2* were identified via tumour testing between 1 January 2016 and 31 December 2021 (n = 7233). Data were collected by chart review. Survival analyses were performed using the Kaplan–Meier method using the log-rank test. (3) Results: A total of 131 patients were identified. The median age was 66. Thirty-three percent of patients had brain metastases. For the *EGFR* cohort, the median OS was 18.6 months for patients who received any systemic therapy (ST) vs. 2.6 months for patients who did not (*p* < 0.001). Median OS was similar for patients treated with ex20ins-specific tyrosine kinase inhibitors (TKIs) vs. other STs (18.6 vs. 15.9 months; *p* = 0.463). The median first-line PFS was 4.1 vs. 7.4 months for patients treated with a TKI vs. other ST (*p* = 0.744). For the *HER2* cohort, the median OS was 9.0 months for patients who received any ST vs. 4.9 months for patients who did not (*p* = 0.015). The median OS was 23.0 months for patients treated with an ex20ins TKI vs. 5.6 months for patients who were not (*p* = 0.019). The median first-line PFS was 5.4 vs. 2.1 months for patients treated with a TKI vs. other ST (*p* = 0.343). (4) Conclusions: Overall survival was significantly longer among ex20ins patients who received any systemic therapy vs. those who did not. Overall survival was significantly better among *HER2* ex20ins patients who received ex20ins-specific TKIs.

## 1. Introduction

Exon 20 insertion mutations (ex20ins) in epidermal growth factor receptor (*EGFR*) is the third most common *EGFR* mutation. They account for approximately 10% of the mutated *EGFR* population and are found in approximately 2% of all non-small-cell lung cancers (NSCLCs) [1,2]. *EGFR* exon 20 insertion mutations are associated with known de novo resistance to most first- and second-generation *EGFR* tyrosine kinase inhibitors (TKIs) [3]. Response rates to third generation TKIs are variable [4,5]. NSCLC patients with *EGFR* ex20ins have a worse prognosis compared with those with classical *EGFR* mutations or other uncommon *EGFR* mutations [6,7]. The most common *EGFR* ex20ins involve amino acids 766–775 in the adjacent loop after the alpha-C helix [2].

Prior to the development of exon 20-targeted agents, the standard of care for *EGFR* ex20ins was similar to NSCLC without driver mutations, namely, involving first-line platinum chemotherapy [8]. In patients with poor performance status who are unable to tolerate standard chemotherapy, the second-generation *EGFR* TKI afatinib was often used; this was based on retrospective data showing objective response rates (ORR) of up to 24% in ex20ins and the post hoc analysis of the original LUX-Lung trials showing afatinib activity in *EGFR* uncommon mutations [9,10]. Recently, novel agents have been developed, including the bispecific antibody amivantamab, targeting the extracellular domain of *EGFR* and *MET*, and the pan-*HER* TKIs mobocertinib and poziotinib, with high affinity for the loop adjacent to the alpha-C helix [2]. Amivantamab has an ORR of 40% in heavily pretreated patients, and the ORR for mobocertinib in pretreated patients is 28% [11,12]. Both drugs have been approved by the FDA for the treatment of *EGFR* exon 20 insertion-mutated lung cancers. Poziotinib is less active in the case of *EGFR* ex20ins compared with its activity in patients with *HER2* ex20ins, with ORRs of 14.8% and 27.8% in previously treated and treatment-naïve patients, respectively [13,14]. Preliminary results from the phase II WU-KONG trial of sunvozertinib therapy in *EGFR* ex20ins lung cancer patients have recently been presented, and these demonstrate a high objective response rate of 73.1% in the first-line setting among 26 evaluable patients [15]. The efficacy data for recently therapeutic agents with exon 20 activity are summarized in Table 1.

Alterations in human epidermal growth factor receptor 2 (*ERBB2*/*HER2*) have been identified as oncogenic drivers in NSCLC. Unlike several other *HER2*-driven cancer types that are primarily due to *HER2* copy number gain/amplifications, approximately 90% of *HER2* alterations in NSCLC are exon 20 insertions that account for 1.5–2% of all NSCLCs [2]. Compared with *EGFR* ex20ins, *HER2* ex20ins are less heterogeneous and commonly involve insertion–duplication of the amino acids YVMA between amino acids 772–780 [16]. In terms of therapeutic strategies, *HER2* ex20ins are less responsive to conventional *HER2* TKIs [17]. Response rates to trastuzumab and pertuzumab given alone, together, or in combination with chemotherapy or to trastuzumab–emtansine (TDM1) are low, based on results from early-phase studies [16,18]. More recently, the novel irreversible pan-*HER* TKI poziotinib has been reported to have clinical activity with an ORR of 28% in pretreated patients [19]. Mobocertinib has been shown to have a robust inhibitory activity against *HER2* ex20ins in preclinical studies [20]; clinical trials evaluating its efficacy against this molecular subtype are currently underway. The antibody–drug conjugate trastuzumab–deruxtecan (T-Dxd) conferred a response rate of 55% in heavily pretreated patients in the Destiny-Lung01 trial and is the only FDA-approved targeted anti-*HER2* therapy for *HER2*-mutated NSCLC [21].

**Table 1:** Summary of selected recently studied agents with exon 20 activity from clinical trials.

	Target	Patient Population	Results
Amivantamab	*EGFR*	Previously treated advanced NSCLC patients with *EGFR* ex20ins (N = 81)	ORR 40%, median PFS 8.3 months, median DoR 11.1 months (CHRYSALIS) [11]
Mobocertinib	*EGFR*	Previously treated advanced NSCLC patients with *EGFR* ex20ins (N = 114)	ORR 28%, median PFS 7.3 months, median DoR 17.5 months, median OS 24 months (EXCLAIM) [12]
Poziotinib	*EGFR* and *HER2*	*EGFR* ex20ins—previously treated advanced NSCLC patients (N = 115)	ORR 14.8%, median PFS 4.2 months, median DoR 7.4 months (ZENITH20-1) [13]
		*EGFR* ex20ins—previously untreated advanced NSCLC patients (N = 79)	ORR 27.8%, median PFS 7.2 months, median DoR 6.5 months (ZENITH20-3) [14]
		*HER2* ex20ins—previously treated advanced NSCLC patients (N = 90)	ORR 27.8%, median PFS 5.5 months, median DoR 5.1 months (ZENITH20-2) [19]
		*HER2* ex20ins—previously untreated advanced NSCLC patients (N = 70)	ORR 41%, median PFS 5.6 months, median DoR 5.7 months (ZENITH20-4) [22]
Sunvozertinib	*EGFR*	Previously untreated advanced NSCLC patients with *EGFR* ex20ins (N = 26 evaluable patients)	ORR 73.1%, median PFS, and DoR not reached (WU-KONG) [15]
T-Dxd	*HER2*	Previously treated advanced NSCLC patients with *HER2* mutations (N = 91 with 78 ex20ins)	ORR 55%, median PFS 8.2 months, median DoR 9.3 months, median OS 17.8 months [21]

ORR, overall response rate; PFS—progression-free survival; DoR—duration of response; OS—overall survival.

Although considerable knowledge has been gained over the last decade with respect to the clinical behaviour, prognosis, and therapeutic options for the classical oncogenic driver mutations in NSCLC, there are limited real-world data characterizing the clinical course among patients harbouring atypical *EGFR* and *HER2* alterations in the evolving therapeutic landscape. Thus, we aimed to characterize the clinical and histopathological features, treatment patterns, and clinical outcomes of *EGFR* and *HER2* ex20ins patients in this retrospective multi-centre real-world analysis of a Canadian NSCLC population.

## 2. Materials and Methods

### 2.1. Study Population

We performed a retrospective chart review of the electronic medical records of patients receiving care across 5 BC Cancer centres across British Columbia, Canada. Eligible patients with a histopathological diagnosis of NSCLC were identified through the Provincial Cancer Genetics Laboratory Database from 1 January 2016 to 31 December 2021 (n = 7233). Patients diagnosed with recurrent or advanced/metastatic NSCLC underwent multi-gene panel-based tumour sequencing via a next-generation sequencing (NGS) technique that included *EGFR* and *HER2* exon 20 mutations.

The inclusion criteria were all of the following: patients identified with exon 20 mutations in *EGFR* or *HER2* during the 6-year study period; patients receiving exon 20-targeted therapies, including those who received therapy through a clinical trial and/or special access programs. Patients with all histology subtypes were included. Exclusion criteria included the following: patients with a T790M mutation; patients with only molecular genetics testing result but no other clinical data (e.g., patients who had tumour testing in British Columbia but who were treated and/or had all diagnostic testing and treatments outside the province. The study protocol was approved by the BC Cancer Research Ethics Board (REB) Accession Number H22-00141.

### 2.2. Data Collection

Patient characteristics from the time of lung cancer diagnosis were retrospectively collected through chart reviews. Information on their PD-L1 status (by immunohistochemistry, using the antibody Dako 22C3) was collected if available. In this analysis, TKIs with exon 20 activity included poziotinib, mobocertinib, and afatinib. Other palliative systemic therapies (STs) included chemotherapy with or without immunotherapy, immunotherapy alone, and gefitinib or osimertinib for patients with *EGFR* ex20ins. Follow-up and survival outcomes were calculated using the start date as the date of diagnosis of advanced/metastatic disease. Overall survival (OS) was defined as the time from diagnosis of advanced/metastatic disease to the time of death, whereas progression-free survival (PFS) was defined as the time from starting systemic therapy to the time of clinical or radiographic progression or death. Time to progression was defined as the time from starting treatment to either radiographic or clinical progression, based on the treating physician’s report. Radiographic imaging was conducted at the treating physician’s discretion. Patients who were alive or who were lost to follow-up at the end of the study period were censored. Adverse events were categorized using definitions as per the Common Terminology Criteria for Adverse Events (CTCAE) version 5.0.

### 2.3. Statistical Analyses

Statistical analyses were performed using SPSS Version 28.0.0. Patient and treatment characteristics were summarized using the median and range for continuous variables, and the count and percentage for categorical variables. OS and PFS were performed using the Kaplan–Meier method using a log-rank test to assess subgroup differences. *p*-values and 95% confidence intervals (CI) were calculated for each variable. A *p*-value of <0.05 was considered as statistically significant.

## 3. Results

**Figure 1:**
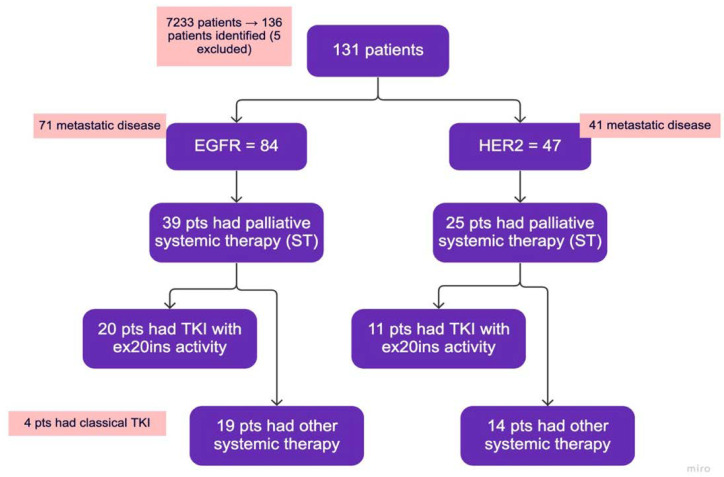
Study flowchart. TKIs with ex20ins activity: poziotinib, mobocertinib, afatinib. Classical TKI: Osimertinib or gefitinib—grouped together with other systemic therapy. Other systemic therapy: chemotherapy with or without immunotherapy, immunotherapy alone, classical TKIs.

Between 1 January 2016 and 31 December 2021, a total of 136 patients were identified. Of these, 5 patients were excluded, and 131 patients were retained for further analysis (Figure 1). Baseline clinical characteristics and demographic data are summarized in Table 2. Out of the 131 patients, 84 had *EGFR* ex20ins, and 47 had *HER2* ex20ins; the median age was 66 years. In the *EGFR* group, 51/84 (61%) were female, 41/84 (49%) were Asian, and 63/84 (75%) had never smoked or were light smokers. In the *HER2* group, 27/47 (57%) were female, 19/47 (40%) were Asian, and 30/47 (64%) had never smoked or were light smokers. A total of 37% of the *EGFR* patients and 26% of the *HER2* patients had brain metastases. Other common sites of metastases included bone, pleura, liver, and the adrenal gland (Table 2). Most patients were diagnosed with de novo metastatic disease.

With respect to histology, 80/84 (95%) of the *EGFR* patients had adenocarcinoma, 3/84 were adenosquamous and 1/84 had a squamous subtype (this patient was a never-smoker). A total of 46/47 (98%) of the *HER2* patients had adenocarcinoma. Concomitant mutations in *EGFR* or *HER2* were rare. Other molecular alterations identified from the NGS panel were uncommon, and these were listed in Table 3A,B. Among *EGFR* ex20ins patients, 21/84 (25%) had tumours with PD-L1 greater or equal to 50%, 25/84 (30%) had 1–49% PD-L1, and 30/84 (36%) had PD-L1 < 1%. Among *HER2* ex20ins patients, 9/47 (19%) had tumours with PD-L1 greater or equal to 50%, 10/47 (11%) had 1–49% PD-L1, and 23/47 (49%) had PD-L1 > 50% (Table 3C).

### 3.1. Treatment Outcomes for Metastatic EGFR ex20ins Patients

**Figure 2:**
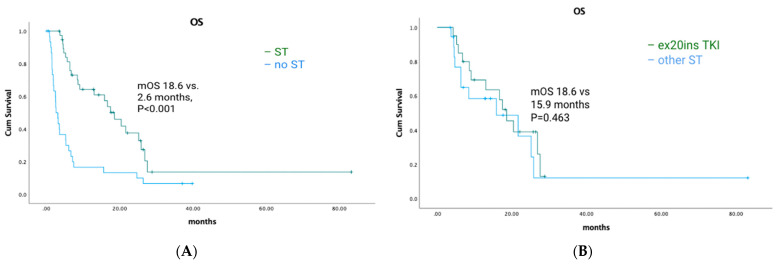
*EGFR*-cohort overall survival and exon 20-targeted therapy. (**A**) Overall survival of patients who received systemic therapy vs. no systemic therapy. (**B**) Overall survival for exon 20 TKIs vs. other systemic therapy.

**Figure 3:**
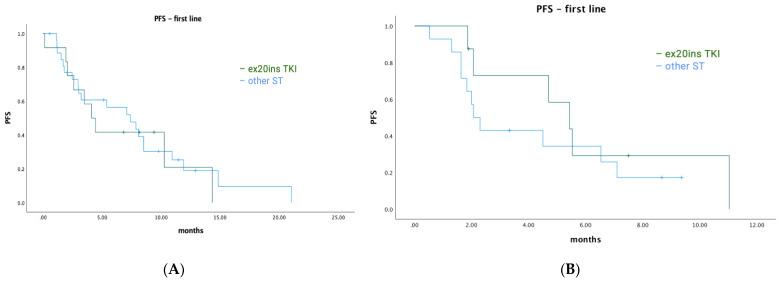
Progression-free survival for first-line systemic therapy. (**A**) *EGFR* cohort—first-line PFS. (**B**) *HER2* cohort—first-line PFS.

**Figure 4:**
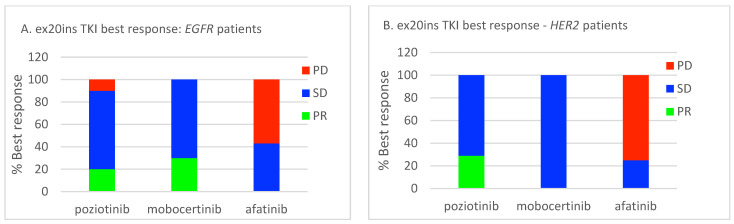
Relative best responses for ex20ins TKIs. (**A**) *EGFR* cohort. (**B**) *HER2* cohort. PR, partial response; SD, stable disease; PD, progressive disease.

Among *EGFR* ex20ins patients, 71/84 had stage IV disease. The median OS for patients with metastatic disease was 7.1 months (95% CI: 4.73–9.54). A total of 39/71 (55%) patients had palliative systemic therapy, of which 20 (51%) received TKIs with activity against ex20ins (i.e., poziotinib, mobocertinib, or afatinib). Ten patients received poziotinib, five patients received mobocertinib, and seven patients received afatinib. Median OS was 18.6 months (95% CI: 13.40–23.80) for patients who received any palliative ST vs. 2.6 months (95% CI: 1.66–3.54) for patients who did not (*p* < 0.001) (Figure 2A). Median OS was similar for patients treated with an ex20ins TKI vs. other ST (18.6 months (95% CI: 13.92–23.28) vs. 15.9 months (95% CI: 0.4–31.73); *p* = 0.463) (Figure 2B). In the first-line setting, median PFS was 7.1 months (95% CI: 2.94–11.26) for all patients; mPFS was 4.1 months (95% CI: 2.52–5.68) vs. 7.4 months (95% CI: 3.64–11.16) for patients treated with a TKI vs. other ST (*p* = 0.744) (Figure 3A). The best responses achieved with the different ex20ins TKIs for *EGFR* patients are shown in Figure 4A. Nine out of 10 patients who received poziotinib and all 5 patients who received mobocertinib had either a partial response or stable disease, whereas 3/7 patients who received afatinib had stable disease. Grade 3 toxicity occurred in 6/39 (15.4%) patients who received systemic therapy and 4/20 (20%) patients received TKIs with exon 20 activity (Table 4).

Among *EGFR* ex20ins patients who received other systemic therapy either in the first-line or post-TKI progression setting, four patients received chemo-immunotherapy with platinum doublet plus pembrolizumab. Eleven patients received immunotherapy as monotherapy. For those who received immunotherapy alone, six received first-line pembrolizumab and five received either nivolumab or pembrolizumab in the second-line or beyond setting. A total of 19 patients received chemotherapy alone. Best response outcomes as well as time to progression are summarized in Table 5A.

Of the 21 *EGFR* ex20ins patients with tumour PD-L1 expression of 50% or higher, 7 patients had received immunotherapy as monotherapy, 5 had received first-line pembrolizumab, and 2 had received pembrolizumab in the second-line or beyond setting. None received chemo-immunotherapy. The median time to progression was 3 months. Four out of seven of these patients had either stable disease or partial response as their best response, whereas three out of seven had progressive disease (Table 5A).

### 3.2. Treatment Outcomes for Metastatic HER2 ex20ins Patients

**Figure 5:**
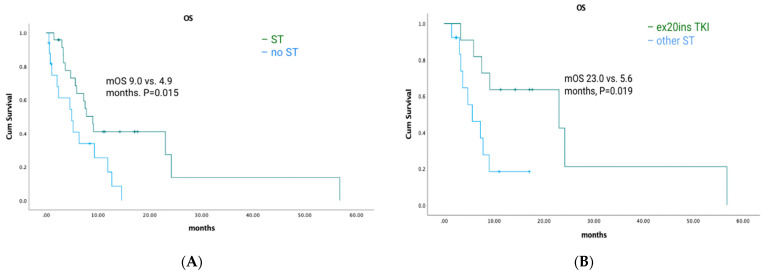
*HER2*-cohort overall survival and exon 20-targeted therapy. (**A**) Overall survival with systemic therapy vs. no systemic therapy. (**B**) Overall survival with an exon 20 TKI vs. other systemic therapy.

Among *HER2* ex20ins patients, 41/47 had stage IV disease. The median OS for patients with metastatic disease was 7.3 months (95% CI: 4.72–9.81). A total of 25/41 (61%) patients had palliative ST, of which 11 (44%) received TKIs with ex20ins activity. Seven patients received poziotinib, one patient received mobocertinib, and four patients received afatinib. The median OS was 9.0 months (95% CI: 6.90–11.16) for patients who received any palliative systemic therapy vs. 4.9 months (95% CI: 1.46–8.40) for patients who did not (*p* = 0.015) (Figure 5A). The median OS was 23.0 months (95% CI: 3.3–48.51) for patients treated with ex20ins TKI vs. 5.6 months (95% CI: 1.58–9.68) for patients who were not (*p* = 0.019) (Figure 5B). In the first-line setting, the median PFS was 4.5 months (95% CI: 0.85–8.16) for all patients who had ST; mPFS was 5.4 months (95% CI: 3.58–7.29) vs. 2.1 months (95% CI: 1.52–2.62) for patients treated with a TKI vs. other ST (*p* = 0.343) (Figure 3B). Best responses achieved with the different ex20ins TKIs for *HER2* patients are shown in Figure 4B. Of the seven patients who received poziotinib, two had a partial response and five had stable disease, and the one patient who received mobocertinib had stable disease, whereas one of the four patients who received afatinib had stable disease. Grade 3 toxicity occurred in 4/25 (16%) patients who received systemic therapy, and in 1/11 (9%) patients who received TKI with exon 20 activity (Table 4).

Among *HER2* ex20ins patients who received other systemic therapy either in the first-line or post-TKI progression setting, nine patients received chemo-immunotherapy with platinum doublet plus pembrolizumab. Seven patients received immunotherapy alone. Among those who received immunotherapy only, two received first-line pembrolizumab, and five received either nivolumab or pembrolizumab in the second-line and beyond setting. A total of 11 patients received chemotherapy alone. The best response as well as time to progression is summarized in Table 5B.

Of the nine *HER2* ex20ins patients with tumour PD-L1 expression of 50% or higher, three patients received immunotherapy as monotherapy: two in first-line and one in third-line therapy. One patient received first-line chemo-immunotherapy. The median time to progression was 2.3 months among those who received immunotherapy as monotherapy. Two out of three patients had progressive disease as their best response and one had stable disease (Table 5B).

## 4. Discussion

Our study is one of the largest population-based real-world cohorts, with 131 patients with *EGFR* and *HER2* ex20ins-mutated advanced/metastatic NSCLC studied over a 6-year time span. The patients captured in this study were treated in the era before newer targeted agents including amivantamab were available, and those who received exon 20-targeted agents were treated either in a clinical trial setting or through patient special-access programs. This study included patients with ex20ins exclusively confirmed via NGS-based panel testing.

The overall survival was 7.1 months for *EGFR*+ patients and 7.3 months for the *HER2* subgroup, which is shorter than what has been reported in other real-world studies [23,24]. This observation is likely explained by several contributing factors specific to the study cohort. A significant proportion of patients (close to 50% of patients in both groups) did not receive palliative systemic therapy following their diagnosis of advanced/metastatic disease, presumably due to their poor performance status and/or rapid clinical deterioration. Among the patients who did receive systemic therapy, the median OS was 18.6 months in the *EGFR* cohort and 9.0 months in the *HER2* cohort, which is similar to what has been previously reported [19,24,25]. In a recently published retrospective review of Canadian NSCLC patients with the three most common *EGFR* driver mutations, the ex20ins cohort had 18 patients, of which 72.2% received systemic therapy; the median OS from the initiation of first-line systemic therapy was 10.5 months [26]. In addition, our study has a significant proportion of patients with brain metastases at the diagnosis of stage IV disease that is both prognostic of clinical outcomes and predictive of the response to standard systemic therapy.

In our study population, *HER2* exon 20 patients who received TKIs with exon 20 activity lived significantly longer compared with those who received other palliative STs. However, this difference was not reproduced in the *EGFR* exon 20 cohort. This is not unexpected as most of the patients who received TKIs with exon 20 activity received poziotinib, and this TKI was shown to have an ORR of 27.8% in the first-line setting and an ORR of 14.8% in pretreated patients with *EGFR* exon 20 mutations [13,14], whereas it was shown to have an ORR of 41% in first-line and 27.8% in pretreated patients with *HER2* exon 20 mutations [19,22]. The different activity of poziotinib against *EGFR* and *HER2* ex20ins was also seen in a prospective study in which patients received poziotinib through the expanded access program [25]. In our report, the observed first-line PFS was 5.4 months in *HER2* patients who received an exon 20 TKI vs. 2.1 months for those who did not. This is very close to the reported PFS of 5.5 months in ZENITH20-2 (the *HER2* cohort). However, given the limitations of the overall small number of patients (N = 11 for those who received exon 20 TKIs), the difference is not statistically significant. Currently, the most promising *HER2*-targeted therapy for NSCLC is T-Dxd, with an ORR of 55% in pretreated patients and a median OS of 17.8 months in the phase II Destiny-Lung01 trial [21]. T-Dxd is not yet funded in most parts of Canada outside of a clinical trial setting for NSCLC patients.

In contrast to the *HER2* cohort, patients with *EGFR* ex20ins who received TKIs with exon 20 activity had a numerically longer but statistically non-significant OS versus those who received other palliative STs. In a recently reported phase II clinical trial, poziotinib was found to have an ORR of 32% and a median PFS of 5.5 months in 50 patients with *EGFR* exon 20 alterations, including both insertions and point mutations [27]. In this trial, poziotinib was shown to have more activity in near-loop insertions (amino acids 767–772) compared with far-loop insertions (amino acids 773–775). Mobocertinib and amivantamab have both been shown to have positive clinical outcomes in pretreated patients with *EGFR* ex20ins and are both approved by the FDA [11,12]. In our patient population, only five patients received mobocertinib and no patients received amivantamab due to lack of access during the study period.

Among the entire study cohort, there were 30 patients with tumour PD-L1 expression levels equal to or greater than 50%, 10 of whom received single-agent immunotherapy; these had a short time to progression, and 5/10 had progressive disease as the best response. These findings are not unexpected as it has been well reported that patients with certain oncogenic driver mutations tend to have a poorer response to immunotherapy in both prospective trials and real-world analyses [28]. In the IMMUNOTARGET registry, 125 patients with sensitizing *EGFR* mutations had an ORR of 12.2% to immune checkpoint inhibitors and a PFS of 2.1 months, whereas 29 patients with *HER2* mutations had an ORR of 7% to immune checkpoint inhibitors and a PFS of 2.5 months [29]. It is unclear whether ex20ins behave similarly to the classic *EGFR* driver mutations as studies are heterogeneous, and rare molecular subtypes are not specifically analyzed. Retrospective data has shown that NSCLC patients with ex20ins were associated with lower PD-L1 expression and tumour mutational burden and a poor response to immunotherapy compared with NSCLC patients without targetable mutations [30]. However, there is also retrospective evidence showing that patients with ex20ins in *EGFR* and *HER2* may derive greater benefit from immunotherapy compared with those carrying classical sensitizing *EGFR* mutations [31]. Further studies are needed to investigate the effect of PD-L1 expression status on the response to immunotherapy in ex20ins patients.

Limitations of our study include the retrospective nature of the review. Although it is a multi-center population-based study, patients are from a single province of Canada. Due to the rarity of the mutations, the sample size for the subgroups is small and may not be sufficient to detect any statistically significant difference. The small sample size also limits further subgroup stratification and detailed analysis of homogeneous groups. Similar to other real-world evidence studies, analyses of data are restricted by descriptive information and, sometimes, missing information, which is largely reliant on the treating physicians and details in the documentations.

In conclusion, we report results from a large population-based study evaluating real-world outcomes for 131 NSCLC patients with exon 20 insertion mutations in *EGFR* and *HER2.* This study reflects the clinical practice and treatment patterns of physicians and clinical outcomes of patients in an era when exon 20 insertion-targeted therapy was either unavailable or had just become available for select patients. Results from this study confirm that, overall, patients with ex20ins have a poor prognosis. Of note, among the *HER2* cohort, overall survival was significantly better for those who received TKIs with exon 20 activity. Our data highlight the ongoing need for improved access to therapeutic agents and clinical trials for lung cancer patients with *EGFR*/*HER2* exon 20 mutations.

## Figures and Tables

**Table 2:** Patient Characteristics.

	*EGFR* (N = 84)	*HER2* (N = 47)
**Age (y), median (range)**	66 (37–87)	67 (27–97)
Sex		
Male	33 (39)	20 (43)
Female	51 (61)	27 (57)
**Ethnicity**		
Asian	41 (49)	19 (40)
Non-Asian	43 (51)	28 (60)
**Smoking status (pack years)**		
Never	47 (56)	26 (55)
Light (</=10)	16 (19)	4 (9)
Heavy (>10)	21 (25)	15 (32)
Unknown	0 (0)	2 (4)
**Stage at initial diagnosis**		
1	6 (7)	2 (4)
2	12 (14)	3 (6)
3	8 (10)	11 (23)
4	58 (69)	31 (67)
**Performance status (ECOG)**		
0	17 (20)	7 (15)
1	38 (45)	26 (55)
2	8 (10)	6 (13)
3	16 (19)	7 (15)
4	5 (6)	1 (2)
**Histology, N (%)**		
Adeno	80 (95)	46 (98)
Adenosquamous	3 (4)	1 (2)
Squamous	1 (1)	0 (0)
**Palliative systemic therapy, N (%)**		
Total who received	39 (46)	25 (53)
Did not receive	45 (54)	22 (47)
# Lines, median (range)	1 (0–5)	1 (0–5)
N who received TKIs	20 (24)	11 (23)
N who received immunotherapy	15 (19)	17 (36)
**Palliative radiation, N (%)**	51 (61)	20 (43)
**Prior curative intent treatment, N (%)**		
Surgery	19 (23)	6 (13)
Radiation	3 (4)	3 (6)
Combined modality	4 (5)	7 (15)
**Sites of metastasis, N (%)**		
Pleura	39 (46)	27 (57)
Liver	18 (21)	9 (19)
Adrenal gland	9 (11)	8 (17)
Bone	46 (55)	23 (49)
Brain	31 (37)	12 (26)

**Table 3:** Molecular Characteristics.

A. *EGFR* Cohort.		
**Exon 20 mutation status**		
Insertion/duplication		83 (99)
Point mutation		1 (1)
**Other concomitant *EGFR* mutations**		
Exon 18 point mutation		2
Exon 21 L858R		1
**Other molecular alterations (N)**		
*CDKN2A*		3
*BRCA1*		2
*BRCA2*		1
*SDHA*		1
*SDHB*		1
*PIK3CA*		2
*NF1*		1
*SMAD4*		1
*PTEN*		1
*MSH2*		1
Overall		12
**B. *HER2* cohort**		
**Exon 20 mutation status**		
Insertion/duplication		47 (100)
**Other molecular alterations (N)**		
*KRAS* G12C		1 (2)
*BRCA1*		1 (2)
*SDHC*		1 (2)
*APC*		1 (2)
*PIK3CA*		1 (2)
*PTEN*		1 (2)
Overall		6 (13)
**C. PD-L1 status**		
**PD-L1**	*EGFR*	*HER2*
<1%	30 (36)	23 (49)
1–49%	25 (30)	10 (21)
50%+	21 (25)	9 (19)
unknown	8 (10)	5 (11)

**Table 4:** Treatment-related adverse events.

	*EGFR*	*HER2*
**All STs**	15.4% (6/39)	16% (4/25)
**ex20ins TKI**	20% (4/20) *	9% (1/11) **

* 2/4 poziotinib—diarrhea, rash; 1/4 mobocertinib—diarrhea; 1/4 afatinib—nausea. ** poziotinib—mucositis.

**Table 5:** Treatment responses with other systemic therapy.

A. *EGFR* Cohort		
Type of Systemic Therapy	Best Response	Time to Progression (Months) (Median, Range)
Chemo-IO (N = 4)	PR (3/4, 75%)	8.6 (3.0–10.5)
	SD (0/4, 0%)	
	PD (1/4, 25%)	
IO monotherapy (N = 11)	PR (3/11, 27%)	3.0 (0.5–21.2)
	SD (1/11, 9%)	
	PD (7/11, 64%)	
Chemotherapy (N = 19)	PR (3/19, 16%)	4.73 (0.5–13.0)
	SD (13/19, 68%)	
	PD (3/19, 16%)	
PD-L1 50% or higher receiving IO monotherapy (N = 7)	PR (3/7, 43%)	3.0 (1.2–21.2)
	SD (1/7, 14%)	
	PD (3/7, 43%)	
**B. *HER2* cohort**		
**Type of Systemic Therapy**	**Best Response**	**Time to Progression (Months)** **(Median, Range)**
Chemo-IO (N = 9)	PR (2/9, 22%)	6.2 (0.5–15.7)
	SD (4/9, 44%)	
	PD (3/9, 33%)	
IO monotherapy (N = 7)	PR (0/7, 0%)	2.3 (0.9–6.0)
	SD (2/7, 29%)	
	PD (5/7, 71%)	
Chemotherapy (N = 11)	PR (1/11, 9%)	2.4 (0.9–8.6)
	SD (5/11, 45%)	
	PD (5/11, 45%)	
PD-L1 50% or higher receiving IO monotherapy (N = 3)	PR (0/3, 0%)	2.3 (1.0–4.5)
	SD (1/3, 33%)	
	PD (2/3, 67%)	

IO, immunotherapy; PR, partial response; SD, stable disease; PD, progressive disease.

## Data Availability

The data presented in this study are available on request from the corresponding author. The data are not publicly available due to sensitive patient information and is in accordance with institutional policy.
